# Supplementary material for: Optimisation of Vitamin B12 Extraction from Green Edible Seaweed (Ulva lactuca) by Applying the Central Composite Design
Source: Molecules. 2022 Jul 12;27(14):4459. doi: 10.3390/molecules27144459 (PMC9319212; doi:10.3390/molecules27144459)
Supplement: Supplementary file 1 [file molecules-27-04459-s001.zip › Supplementary Data/Supplementary Table S1_The concentrations of CN-Cbl obtained through the boiling, orbital shaking and ultrasonic assisted extraction.pdf]

Supplementary Table S1: The concentrations of CN-Cbl obtained through the boiling, orbital shaking and ultrasonic assisted extraction methods

**Run**	**Parameters**			**Conconetration of CN-Cbl (mg/mL)**											
	**Solvent:solvent ratio ($MeOH:H_2O$)**	**pH**	**Solute:solvent ratio (g/mL)**	**Boiling**				**Orbital Shaking**				**Ultrasonic assisted**			
				**ODB**	**SDB**	**ADB**	**FDB**	**ODO**	**SDO**	**ADO**	**FDO**	**ODU**	**SDU**	**ADU**	**FDU**
**1**	25:75	3	3:60	0.0155	0.0090	0.0122	0.0067	0.0265	0.0155	0.0170	0.0178	0.0224	0.0131	0.0153	0.0167
**2**	25:75	3	3:60	0.0210	0.0093	0.0075	0.0066	0.0170	0.0261	0.0241	0.0245	0.0236	0.0159	0.0146	0.0148
**3**	75:25	3	3:60	0.0079	0.0049	0.0046	0.0075	0.0085	0.0257	0.0108	0.0106	0.0015	0.0207	0.0168	0.0106
**4**	75:25	3	3:60	0.0083	0.0084	0.0087	0.0054	0.0220	0.0234	0.0095	0.0139	0.0015	0.0134	0.0175	0.0133
**5**	25:75	5	3:60	0.0160	0.0075	0.0087	0.0067	0.0311	0.0176	0.0133	0.0203	0.0100	0.0116	0.0103	0.0107
**6**	25:75	5	3:60	0.0174	0.0079	0.0089	0.0068	0.0210	0.0176	0.0200	0.0262	0.0102	0.0176	0.0098	0.0153
**7**	75:25	5	3:60	0.0126	0.0071	0.0129	0.0042	0.0114	0.0159	0.0108	0.0126	0.0103	0.0145	0.0101	0.0136
**8**	75:25	5	3:60	0.0151	0.0062	0.0109	0.0056	0.0164	0.0142	0.0140	0.0272	0.0124	0.0112	0.0129	0.0092
**9**	25:75	3	3:90	0.0099	0.0071	0.0060	0.0034	0.0146	0.0246	0.0131	0.0156	0.0172	0.0107	0.0136	0.0154
**10**	25:75	3	3:90	0.0084	0.0049	0.0036	0.0053	0.0121	0.0356	0.0106	0.0185	0.0163	0.0123	0.0106	0.0165
**11**	75:25	3	3:90	0.0172	0.0089	0.0065	0.0079	0.0079	0.0268	0.0170	0.0156	0.0135	0.0175	0.0080	0.0143
**12**	75:25	3	3:90	0.0127	0.0093	0.0093	0.0056	0.0087	0.0155	0.0173	0.0158	0.0161	0.0151	0.0093	0.0154
**13**	25:75	5	3:90	0.0090	0.0038	0.0108	0.0031	0.0321	0.0161	0.0245	0.0253	0.0018	0.0160	0.0075	0.0089
**14**	25:75	5	3:90	0.0098	0.0050	0.0064	0.0046	0.0064	0.0113	0.0124	0.0244	0.0015	0.0163	0.0104	0.0091
**15**	75:25	5	3:90	0.0186	0.0068	0.0159	0.0055	0.0286	0.0138	0.0122	0.0178	0.0146	0.0098	0.0134	0.0118
**16**	75:25	5	3:90	0.0193	0.0088	0.0160	0.0080	0.0114	0.0155	0.0120	0.0215	0.0173	0.0155	0.0130	0.0108
**17**	50:50	4	3:75	0.0138	0.0059	0.0065	0.0051	0.0103	0.0295	0.0159	0.0181	0.0167	0.0125	0.0159	0.0171
**18**	50:50	4	3:75	0.0131	0.0075	0.0058	0.0066	0.0119	0.0289	0.0135	0.0198	0.0150	0.0099	0.0145	0.0197
**19**	50:50	4	3:75	0.0188	0.0055	0.0071	0.0069	0.0190	0.0307	0.0159	0.0227	0.0178	0.0117	0.0139	0.0191
**20**	50:50	4	3:75	0.0144	0.0110	0.0118	0.0054	0.0133	0.0192	0.0229	0.0127	0.0181	0.0092	0.0137	0.0167
**21**	50:50	4	3:75	0.0140	0.0104	0.0093	0.0065	0.0157	0.0155	0.0275	0.0174	0.0157	0.0105	0.0126	0.0173

ODB: Oven-dried, extracted by boiling method
SDB: Sun-dried sample, extracted by boiling method
ADB: Air-dried sample, extracted by boiling method
FDB: Freeze-dried, extracted by boiling method
ODO: Oven-dried, extracted by orbital shaking method
SDO: Sun-dried, extracted by orbital shaking method
ADO: Air-dried, extracted by orbital shaking method
FDO: Freeze-dried, extracted by orbital shaking method
ODU: Oven-dried, extracted by ultrasonic-assisted extraction method
SDU: Sun-dried, extracted by ultrasonic-assisted extraction method
ADU: Air-dried, extracted by ultrasonic-assisted extraction method
FDU: Freeze-dried, extracted by ultrasonic-assisted extraction method
