# Supplementary material for: Optimisation of Vitamin B12 Extraction from Green Edible Seaweed (Ulva lactuca) by Applying the Central Composite Design
Source: Molecules. 2022 Jul 12;27(14):4459. doi: 10.3390/molecules27144459 (PMC9319212; doi:10.3390/molecules27144459)
Supplement: Supplementary file 1 [file molecules-27-04459-s001.zip › Supplementary Data/Supplementary Table S6_Table of experimental run for the validation of CCD.pdf]

Supplementary Table S6: Table of experimental run for the validation of CCD

Response	Predicted (mg/100 g dw)	Actual (mg/100 g dw)
Conc. of CN-Cbl	3.5743	3.1617

95 % PI = 95 % prediction interval; Two-sided Confidence = 95%
