# Supplementary material for: Optimisation of Vitamin B12 Extraction from Green Edible Seaweed (Ulva lactuca) by Applying the Central Composite Design
Source: Molecules. 2022 Jul 12;27(14):4459. doi: 10.3390/molecules27144459 (PMC9319212; doi:10.3390/molecules27144459)
Supplement: Supplementary file 1 [file molecules-27-04459-s001.zip › Supplementary Data/Supplementary Table S2_Table of ANOVA for the [Cn-Cbl] extracted from ODB using.pdf]

Supplementary Table S2: Table of ANOVA for the [Cn-Cbl] extracted from ODB using 2-Level Factorial

Source	Sum of Squares	df	Mean Square	F-value	p-value	
**Model**	0.0003	7	1539.82	22.26	< 0.0001	significant
A-Solvent:solvent ratio (MeOH:H_2O)	57.02	1	57.02	0.8245	0.3852	
B-pH	1086.47	1	1086.47	15.71	0.0027	
C-Solute:solvent ratio	161.96	1	161.96	2.34	0.1569	
AB	1117.35	1	1117.35	16.16	0.0024	
AC	7713.28	1	7713.28	111.52	< 0.0001	
BC	192.03	1	192.03	2.78	0.1266	
ABC	450.64	1	450.64	6.52	0.0287	
Curvature	156.36	1	156.36	2.26	0.1636	
**Pure Error**	691.62	10	69.16			
**Cor Total**	11626.73	18				
