# Supplementary material for: Optimisation of Vitamin B12 Extraction from Green Edible Seaweed (Ulva lactuca) by Applying the Central Composite Design
Source: Molecules. 2022 Jul 12;27(14):4459. doi: 10.3390/molecules27144459 (PMC9319212; doi:10.3390/molecules27144459)
Supplement: Supplementary file 1 [file molecules-27-04459-s001.zip › Supplementary Data/Supplementary Table S4_Table of ANOVA for the [Cn-Cbl] extracted from ODU using.pdf]

Supplementary Table S4: Table of ANOVA for the [Cn-Cbl] extracted from ODU using 2-Level Factorial

Source	Sum of Squares	df	Mean Square	F-value	p-value	
**Model**	0.0008	7	0.0001	76.53	< 0.0001	significant
A-Solvent:solvent ratio (MeOH:H2O)	0.0000	1	0.0000	10.53	0.0070	
B-pH	0.0001	1	0.0001	49.69	< 0.0001	
C-Solute:solvent ratio	2.448E-06	1	2.448E-06	1.69	0.2180	
AB	0.0004	1	0.0004	261.72	< 0.0001	
AC	0.0003	1	0.0003	184.21	< 0.0001	
BC	0.0000	1	0.0000	20.66	0.0007	
ABC	0.0000	1	0.0000	7.22	0.0198	
Curvature	0.0001	1	0.0001	60.40	< 0.0001	
**Pure Error**	0.0000	12	1.448E-06			
**Cor Total**	0.0009	20				
