# Supplementary material for: Optimisation of Vitamin B12 Extraction from Green Edible Seaweed (Ulva lactuca) by Applying the Central Composite Design
Source: Molecules. 2022 Jul 12;27(14):4459. doi: 10.3390/molecules27144459 (PMC9319212; doi:10.3390/molecules27144459)
Supplement: Supplementary file 1 [file molecules-27-04459-s001.zip › Supplementary Data/Supplementary Table S5_ Table of ANOVA for the [Cn-Cbl] extracted from ADU using.pdf]

Supplementary Table S5: Table of ANOVA for the [Cn-Cbl] extracted from ADU using 2-Level Factorial

Source	Sum of Squares	df	Mean Square	F-value	p-value	
**Model**	0.0001	7	0.0000	10.49	0.0003	significant
A-Solvent:solvent ratio (MeOH:H2O)	4.951E-06	1	4.951E-06	2.97	0.1105	
B-pH	0.0000	1	0.0000	12.55	0.0041	
C-Solute:solvent ratio	0.0000	1	0.0000	17.32	0.0013	
AB	0.0000	1	0.0000	7.24	0.0196	
AC	2.031E-06	1	2.031E-06	1.22	0.2915	
BC	0.0000	1	0.0000	21.41	0.0006	
ABC	0.0000	1	0.0000	10.70	0.0067	
Curvature	0.0000	1	0.0000	9.61	0.0092	
**Pure Error**	0.0000	12	1.668E-06			
**Cor Total**	0.0002	20				
