# Supplementary material for: Optimisation of Vitamin B12 Extraction from Green Edible Seaweed (Ulva lactuca) by Applying the Central Composite Design
Source: Molecules. 2022 Jul 12;27(14):4459. doi: 10.3390/molecules27144459 (PMC9319212; doi:10.3390/molecules27144459)
Supplement: Supplementary file 1 [file molecules-27-04459-s001.zip › Supplementary Data/Supplementary Table S3_Table of ANOVA for the [Cn-Cbl] extracted from ADB using.pdf]

Supplementary Table S3: Table of ANOVA for the [Cn-Cbl] extracted from ADB using 2-Level Factorial

Source	Sum of Squares	df	Mean Square	F-value	p-value	
**Model**	0.0002	7	0.0000	4.57	0.0106	significant
A-Solvent:solvent ratio (MeOH:H2O)	0.0000	1	0.0000	5.27	0.0406	
B-pH	0.0001	1	0.0001	12.69	0.0039	
C-Solute:solvent ratio	2.563E-10	1	2.563E-10	0.0001	0.9945	
AB	0.0000	1	0.0000	5.45	0.0378	
AC	0.0000	1	0.0000	5.57	0.0361	
BC	0.0000	1	0.0000	2.82	0.1186	
ABC	9.521E-07	1	9.521E-07	0.1859	0.6740	
Curvature	5.488E-06	1	5.488E-06	1.07	0.3210	
**Pure Error**	0.0001	12	5.120E-06			
**Cor Total**	0.0002	20				
